# Identification of epidermal growth factor receptor-positive glioblastoma using lipid-encapsulated targeted superparamagnetic iron oxide nanoparticles in vitro

**DOI:** 10.1186/s12951-017-0313-2

**Published:** 2017-11-22

**Authors:** Huai-Lu Chen, Fei-Ting Hsu, Yu-Chieh Jill Kao, Hua-Shan Liu, Wan-Zhen Huang, Chia-Feng Lu, Ping-Huei Tsai, Ahmed Atef Ahmed Ali, Gilbert Aaron Lee, Ray-Jade Chen, Cheng-Yu Chen

**Affiliations:** 1 Translational Imaging Research Center, College of Medicine, Taipei Medical University, Taipei, Taiwan; 2 Department of Medical Research, Taipei Medical University Hospital, Taipei Medical University, Taipei, Taiwan; 3 Department of Medical Imaging, Taipei Medical University Hospital, Taipei Medical University, Taipei, Taiwan; 4 School of Biomedical Engineering, College of Biomedical Engineering, Taipei Medical University, Taipei, Taiwan; 5 Department of Radiology, School of Medicine, College of Medicine, Taipei Medical University, Taipei, Taiwan; 6 Department of Biomedical Imaging and Radiological Sciences, National Yang-Ming University, Taipei, Taiwan; 7 Department of Surgery, School of Medicine, College of Medicine, Taipei Medical University, Taipei, Taiwan; 8 Division of General Surgery, Department of Surgery, Taipei Medical University Hospital, Taipei, Taiwan

**Keywords:** Epidermal growth factor receptor (EGFR), Targeted superparamagnetic iron oxide (SPIO) nanoparticle, Magnetic resonance imaging (MRI), Glioblastoma, Lipid-encapsulated nanoparticle

## Abstract

**Background:**

Targeted superparamagnetic iron oxide (SPIO) nanoparticles have emerged as a promising biomarker detection tool for molecular magnetic resonance (MR) image diagnosis. To identify patients who could benefit from Epidermal growth factor receptor (EGFR)-targeted therapies, we introduce lipid-encapsulated SPIO nanoparticles and hypothesized that anti-EGFR antibody cetuximab conjugated of such nanoparticles can be used to identify EGFR-positive glioblastomas in non-invasive T_2_ MR image assays. The newly introduced lipid-coated SPIOs, which imitate biological cell surface and thus inherited innate nonfouling property, were utilized to reduce nonspecific binding to off-targeted cells and prevent agglomeration that commonly occurs in nanoparticles.

**Results:**

The synthesized targeted EGFR-antibody-conjugated SPIO (EGFR-SPIO) nanoparticles were characterized using dynamic light scattering, zeta potential assays, gel electrophoresis mobility shift assays, transmission electron microscopy (TEM) images, and cell line affinity assays, and the results showed that the conjugation was successful. The targeting efficiency of the synthesized EGFR-SPIO nanoparticles was confirmed through Prussian blue staining and TEM images by using glioblastoma cell lines with high or low EGFR expression levels. The EGFR-SPIO nanoparticles preferentially targeted U-251 cells, which have high EGFR expression, and were internalized by cells in a prolonged incubation condition. Moreover, the T_2_ MR relaxation time of EGFR-SPIO nanoparticles could be used for successfully identifying glioblastoma cells with elevated EGFR expression in vitro and distinguishing U-251 cells from U-87MG cells, which have low EFGR expression.

**Conclusion:**

These findings reveal that the lipid-encapsulated EGFR-SPIO nanoparticles can specifically target cells with elevated EGFR expression in the three tested human glioblastoma cell lines. The results of this study can be used for noninvasive molecular MR image diagnosis in the future.

**Electronic supplementary material:**

The online version of this article (10.1186/s12951-017-0313-2) contains supplementary material, which is available to authorized users.

## Background

Every year, approximately 250,000 individuals are diagnosed with brain and nervous tumors worldwide [1]. Among these tumors, glioblastoma (formerly glioblastoma multiforme; GBM) is the most common and lethal [1]. In the United States alone, approximately 18,000 people are diagnosed with GBM annually, and GBM accounts for approximately 13,000 deaths every year [2]. Despite the advances in treatments including intensive surgery resection, radiation, and chemotherapy that successfully control the disease progression in some other cancers, the median survival of GBM patients remains 12–15 months with a 5-year survival rate of less than 5% [3, 4]. Therefore, a novel method of improving the efficacy of GBM retreatment is urgently required.

A strategy for improving the treatment outcome is identifying and targeting tumor-specific markers or gene abnormalities present in tumors. A common genetic abnormality occurring in approximately 57.4% [5] of GBM patients is amplification of the epidermal growth factor receptor (EGFR) [6]. The EGFR is a member of the ErbB family of receptors, which consists of four receptor tyrosine kinases: EGFR (ErbB1, Her1), ErbB2 (Neu, Her2), ErbB3 (Her3), and ErbB4 (Her4) [7]. The binding of ligands to their ectodomain of the receptor promotes homodimer and heterodimer formation between receptors [8], which is essential for the activation of the intracellular tyrosine kinase domain and phosphorylation of the C-terminal tail [9]. These signaling processes influence downstream cellular processes, including cell proliferation, survival, angiogenesis, metabolism, and cell differentiation.

Epidermal growth factor receptor abnormality enhances growth, migration, angiogenesis, and metastatic progression in solid tumors [10]. Moreover, EGFR overexpression is a poor prognostic factor and is correlated with decreased overall survival in GBM patients [11, 12]. In addition, constitutive activation of EGFRvIII mutation has been frequently detected in solid tumors, including GBM and breast cancer [13, 14]. The expression of EGFRvIII was limited in normal tissues [15]. Moreover, a significant fraction of overexpressed EGFR in glioma is the constitutively active variant EGFRvIII form [16], which makes it a suitable marker for targeting GBM cells.

Several monoclonal antibody (mAb) drugs including cetuximab, panitumumab, and nimotuzumab [17, 18] have been developed to target both wtEGFR and EGFRvIII for modeling malignant diseases caused by EGFR abnormality. Despite mAb-based EGFR-targeted therapy being successful for melanoma, renal cell carcinoma, and hematologic cancers, the effectiveness of such mAb drugs in GBM treatment remains to be elucidated. The treatment of EGFR-amplified GBM cells with cetuximab in subcutaneous and intracranial mouse xenografts resulted in a significant decrease in proliferation and an increase in overall survival and apoptosis [19]. Moreover, clinical trials using a combination therapy of nimotuzumab and radiotherapy reported increased viability in GBM patients [18].

To identify patients who may benefit from EGFR-targeted therapy, evaluating the EGFR status and its distribution is critical before administering targeted therapies. Conventionally, the EGFR status of tumors is identified using fine-needle biopsy or specimens from surgical resection. However, a noninvasive, systematic approach for EGFR status examination is unavailable and must be developed.

Noninvasive molecular magnetic resonance imaging (MRI) has recently emerged as a diagnostic method that may help in overcoming some difficulties encountered in treating GBMs [20]. Superparamagnetic iron oxide (SPIO) nanoparticles are the most commonly investigated MRI contrast agents [21–23] and can be visualized in T_2_ MRI sequences as a hypointense signal (dark, negative contrast enhancement) [24]. By contrast, gadolinium can be visualized in T_1_ MRI sequences as a hyperintense signal (bright, positive contrast) [25, 26].

In addition to passive sorption by tumor cells, the conjugation of SPIO nanoparticles to tumor-specific ligands, including antibodies, peptides, and therapeutic compounds, has been reported to efficiently enhance MR image contrast for selected targets [20, 27]. Among available conjugates, monoclonal antibodies provide the highest affinity toward their ligands and are thus an ideal candidate for conjugation to targeted SPIO nanoparticles.

Conventionally, nanoparticle surface functionalization groups such as dextran, heparin, and dimercaptosuccinic acid and the net charge of SPIO nanoparticles markedly influence the nontargeted internalization of SPIO nanoparticles by cancer cells [28]. Recent studies have reported that nonfouling lipid-coated surfaces can efficiently reduce nonspecific binding to synthesized ex vivo surfaces [29, 30]. However, the potential of using lipid-encapsulated SPIO nanoparticles in MRI for GBM diagnosis has not been fully investigated.

Thus, this study explored the potential of employing lipid-encapsulated SPIO nanoparticles for detecting EGFR-overexpressing GBM cells by EGFR targeting cetuximab. We hypothesized that lipid-encapsulated SPIO nanoparticles conjugated with the anti-EGFR mAb cetuximab could specifically target EGFR-positive GBM cells and generate distinguishable molecular MR images for noninvasive EGFR detection.

## Methods

### Antibodies and reagents

Unless otherwise mentioned, all supplies were purchased from Sigma-Aldrich (St. Louis, MO, USA). The antibodies used in this study were the anti-EGFR antibody cetuximab (Merk Serono, Taiwan), the anti-EGFR-AF594 antibody (Cell Signaling Technology, Danvers, MA, USA, #8742), and Alexa-488-conjugated goat anti-rabbit and goat anti-human immunoglobulin (IgG) secondary antibodies (Thermo Fisher Scientific, Waltham, MA USA). The reagents were sulfosuccinimidyl 4-(N-maleimidomethyl) cyclohexane-1-carboxylate (sulfo-SMCC), 2-iminothiolane (Thermo Fisher Scientific, Waltham, MA, USA), 6–10-nm non-coated amino-terminated SPIOs (Taiwan Advanced Nanotech Inc., Taiwan; TANBead USPIO-101) and 10-nm lipid-based SPIO nanoparticles with amine (Ocean NanoTech, San Diego, CA, USA, #ILA-10).

### Cell culture and MTT cell metabolism activity assay

The human GBM cell line U-87MG and DBTRG-05MG were obtained from the Bioresource Collection and Research Center (Hsinchu, Taiwan), and U-251 was provided by Dr. Yung-Hsiao Chiang. Tissue culture media and reagents were purchased from Gibco (Thermo Fisher Scientific, Waltham, MA, USA). An MTT metabolism activity assay was performed to evaluate the viability of tested cells. Cultured cells were incubated in culture media containing 0.5 mg/ml thiazolyl blue tetrazolium bromide for 2 h and then dissolved in 100 μl of DMSO for optical density measurement at 595 nm.

### EGFR-SPIO nanoparticles construction

The EGFR-SPIO nanoparticles are constructed as described previously [31] in a three steps manner. The lipid-coated SPIO nanoparticles 10 nm in size were purchased from OceanNanoTech Inc. Briefly, 1 mg of amine-functionalized lipid-coated SPIO nanoparticles [concentration: 1 mg/ml (Fe) = 0.86 nmol/ml (nanoparticles)] 10 nm in size were first reacted with 150 μl/ml sulfo-SMCC (10 mg/ml) at room temperature for 1 h to obtain maleimide-functionalized SPIO nanoparticles. Second, 1.29 nmol of the anti-EGFR antibody cetuximab was treated with 35.6 μl iminothiolane (10 mg/ml) at room temperature for 30 min. Third, the antibody and maleimide-lipid-coated SPIO nanoparticles were cleaned up by 10 K Nanosep filter (Pall Corporation; Port Washington, NY, USA), mixed, and reacted at 4 °C overnight. The unused maleimide-functionalized groups were then blocked by excess cystein for 15 min at room temperature. The antibody-conjugated SPIO nanoparticles were separated using an MS column (Miltenyi Biotech, Germany) and washed with sterilized phosphate-buffered saline (PBS) at a volume > 25 times greater than the column bed volume to remove unconjugated antibodies. The number of immobilized EGFR antibody molecules per SPIO nanoparticle was estimated to be 1.5 based on the molarities of components in the reaction.

### Dynamic light scattering and zeta potential measurement

To determine the nanoparticle size distribution and zeta potential, dynamic light scattering (DLS) measurement was performed using the Zetasizer Nano ZS (Malvern Instruments, UK) according to the manufacturer’s instructions.

### Iron concentration measurement through colorimetric ferrozine assay

A colorimetric ferrozine assay was used to measure the iron concentration of SPIO nanoparticles conjugated with an antibody or adsorbed by cells as described previously [32]. In brief, 1.5 × 10^5^ of cells were seeded on 24 well plate. After cells were attached, culture media were removed and replaced with media containing indicated iron oxide reagents. To measure the iron concentration, the cells were washed with PBS three times before dissociated by 100 μl of iron-releasing reagent [freshly mixed equal volumes of 1.4 M HCl and 4.5% (w/v) KMnO_4_] for 2 h at 60 °C. After cooling the mixture to room temperature, 30 μl of iron-detection reagent (6.5 mM ferrozine, 6.5 mM neocuproine, 2.5 M ammonium acetate, and 1 M ascorbic acid dissolved in water) was added to each sample. After 30 min, 100 μl of the solution in each tube was transfer to a 96-well plate and measured for optical density at 595 nm on a microplate reader.

### Immunofluorescence staining and flow cytometry assay

Immunofluorescence (IF) staining and flow cytometry analyses were performed according to previously described procedures [33]. Single-cell suspensions of indicated cells were subsequently analyzed using the Attune acoustic focusing cytometer (Applied Biosystems, Foster City, CA, USA). EGFR positive cells were defended as cells with signal intensity above 1/1000 threshold of isotype control. IF images of stained cells were acquired using the Nikon Eclipse Ti-U microscope system.

### Prussian blue staining and transmission electron microscopy for iron oxide nanoparticles

In brief, the prepared slides were immersed in a 2% solution containing potassium ferrocyanide and hydrochloric acid at equal volumes (1:1) for 20 min for Prussian blue staining. After three distilled H_2_O washes, cells were counterstained by incubating the slides in neutral fast red solution for 10 min before two final H_2_O washes. Transmission electron microscopy (TEM) images were acquired using the HT-7700 microscope (Hitachi, Japan) according to the manufacturer’s instructions.

### Western blot

A western blot analysis was performed as previously described [34]. In brief, the total protein of the harvested cells was extracted with lysis buffer containing protease inhibitors, and the concentration was measured using the BCA protein assay. Subsequently, sodium dodecyl sulfate polyacrylamide gel electrophoresis was performed, and the samples were transferred to polyvinylidene difluoride (PVDF) membranes. The membranes were then probed with indicated primary antibodies and horseradish peroxidase (HRP) secondary antibodies. Enhanced chemiluminescence (ECL) signals were examined and quantified using the Bio-Rad ChemiDoc Touch imaging system.

### MRI measurements

MR images were obtained from the 7T Bruker PharmaScan MRI scanner by using a volume coil with an inner diameter of 72 mm (Bruker BioSpin, MA, USA). T_2_-weighted images were acquired using spin-echo sequences with an echo time (TE) of 8 ms, a repetition time (TR) of 3000 ms, 40 echoes, a flip angle of 90°, a field of view of 50 × 50 mm, a resolution of 256 × 256, and a slice thickness of 0.5 mm. The MRI samples were EGFR-antibody-functionalized SPIO (EGFR-SPIO) nanoparticle phantoms and cultured cells treated with SPIO nanoparticles suspended in 1% agarose gel.

## Results

### EGFR expression profile in human GBM cell lines

To identify tumor cells with elevated EGFR expression in heterogeneous GBMs and select suitable models for evaluating targeting efficiency, the human GBM cell lines U-87MG, U-251, and DBTRG-05MG were used for evaluating the EGFR expression profile through Western blotting, flow cytometry, and IF staining.

**Fig. 1:**
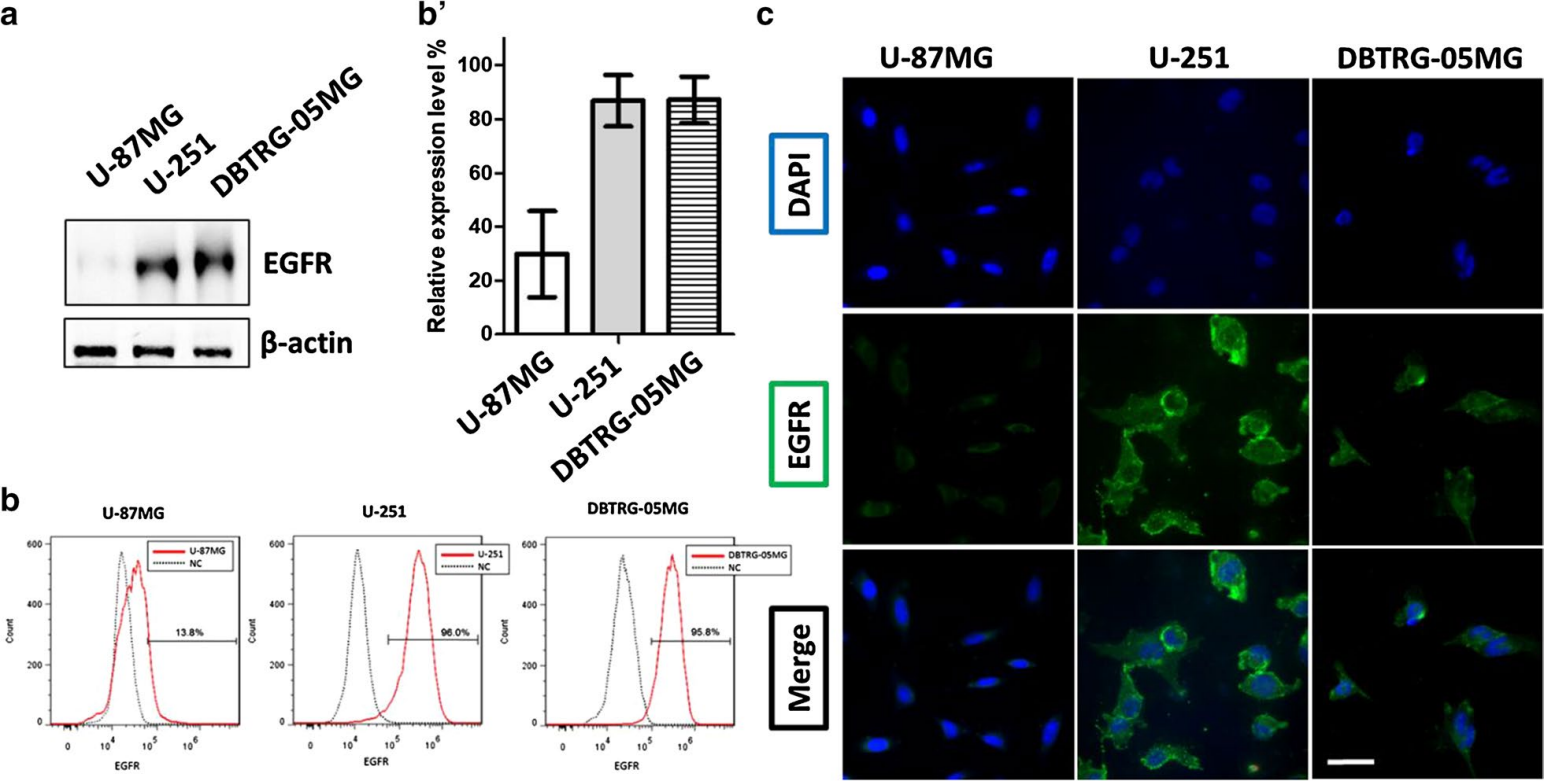
Expression of EGFR in GBM cell lines. **a** Western blot analysis of EGFR level in GBM cell lines. Cell lysates obtained from the U-87MG, U-251, and DBTRG-05MG GBM cell lines were subjected to Western blot analysis with the anti-EGFR antibody. The β-actin level served as a loading control. **b** Flow cytometry analysis of EGFR expression profile in untreated GBM cells. The indicated cells were collected and labeled the with anti-EGFR antibody and Alexa Fluore-488 secondary antibody to determine the EGFR level. Cells with EGFR signals that were more than 0.1% stronger than those of the antibody control were categorized as EGFR-positive cells, and the percentage of positive cells is expressed as the relative EGFR expression level. **b’** EGFR-positive cells quantified using flow cytometry (*n* = 3). **c** IF staining of EGFR-expressing cells. The indicated GBM cells were stained with anti-EGFR (green) and DAPI (blue). Native EGFR was detected in U-251 and DBTRG-05MG cells. Green, anti-EGFR; blue, DAPI. Scale bar, 20 µm

The Western blot results revealed detectable EGFR expression in U-87MG, U-251, and DBTRG-05MG cells, which suggested that all these cells expressed the EGFR. The band intensity of EGFR expression was 34.23-fold higher in U-251 and 53.74-fold higher in DBTRG-05MG compared with the band intensity in U-87MG (Fig. 1a). To further evaluate the EGFR expression profile in its native form, flow cytometry analysis and IF staining were performed. Flow cytometry analysis indicated that U-251 and DBTRG-05MG had 86.80 ± 16.52% and 87.18 ± 14.77% cells detected EGFR positive, which are significantly higher compared with the U-87MG (29.80 ± 15.95%), as detected using the anti-EGFR monoclonal antibody cetuximab (Fig. 1b). In addition, consistent with the Western Blot and flow cytometry results, IF staining analysis demonstrated a weak signal from U-87MG (Fig. 1c, left), but strong signals from U-251 and DBTRG-05MG (Fig. 1c, middle, right) when they were stained with cetuximab. These results indicate that U-251 and DBTRG-05MG have elevated EGFR levels (EGFR^high^) compared with U-87MG (EGFR^low^).

### Characterization of lipid-encapsulated and uncoated SPIO nanoparticles

To enhance the signal-to-noise ratio for MR images, minimizing the nonspecific absorption of SPIO nanoparticles is essential. Lipid surface modification, in which mimic the cell surface consisted with lipid, has been reported to be an efficient nonfouling strategy to reduce nonspecific binding in the complex hematopoietic environment [29, 30]. To select the SPIO nanoparticles for targeted colloidal synthesis, uncoated SPIO nanoparticles and lipid-encapsulated SPIO nanoparticles were characterized and their absorbance rates were compared in physical conditions.

**Fig. 2:**
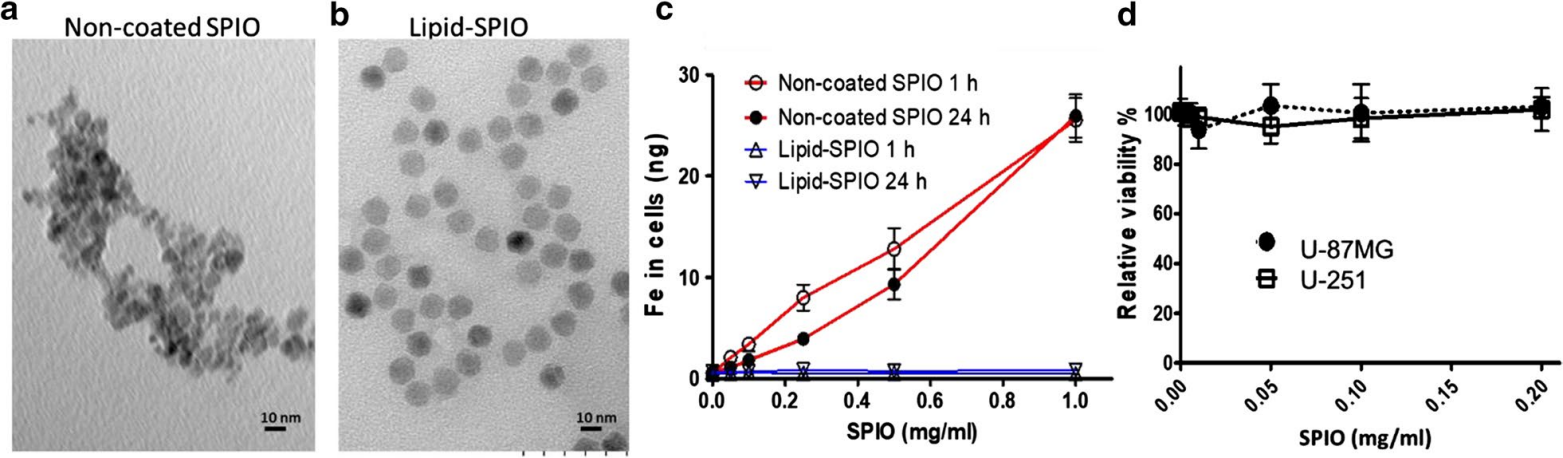
Lipid-coated SPIO nanoparticles exhibit superior reduced nonspecific uptake rate compared with uncoated SPIO nanoparticles. TEM images of **a** non-coated SPIO nanoparticles and **b** lipid-encapsulated SPIO nanoparticles acquired from a different manufacturer. Scale bar, 10 nm. **c** Quantification of adsorption rate for non-coated SPIO and lipid-encapsulated SPIO nanoparticles in the U-87MG cell line. Indicated nanoparticles were added to the U-87MG culture medium at final concentrations of 1, 0.5, 0.25, 0.1, 0.05, 0.025, 0.01, and 0 mg/ml before incubating the cells at 37 °C for 1 or 24 h. The cells were then subjected to a colorimetric ferrozine assay to measure the Fe concentration. **d** The toxicity of lipid-encapsulated SPIO nanoparticles to U-87MG and U-251 cells was evaluated using an MTT viability assay. Lipid-encapsulated SPIO nanoparticles with final Fe concentrations of 0.2, 0.1, 0.05, 0.01, and 0 μg/ml in culture media were incubated with the indicated cells for 24 h before subjecting them to the MTT assay. Error bars, SEM

The morphology of uncoated SPIO and lipid-encapsulated SPIO nanoparticles was first evaluated through TEM. The images demonstrated that the 6–10-nm uncoated SPIO nanoparticles formed clumps > 30 nm when dissolved in PBS at pH 7.4 during preparation (Fig. 2a). By contrast, the TEM images of 10-nm lipid-encapsulated SPIO nanoparticles acquired from a different manufacturer revealed a distinctly separated morphology in the same condition (Fig. 2b), indicating that lipid encapsulation provides an efficient nonfouling property to prevent SPIO nanoparticles from agglomerating.

Furthermore, the nonspecific sorption of uncoated and lipid-encapsulated SPIO nanoparticles was evaluated in a GBM cell line. U-87MG cells were incubated in culture media containing uncoated or lipid-encapsulated SPIO nanoparticles with 1, 0.5, 0.25, 0.1, 0.05, 0.025, 0.01, and 0 mg/ml iron at 37 °C for 1 or 24 h. After washes with PBS, the cells were lysed and subjected to a ferrozine assay to measure the total Fe content present in each well. Remarkably, the lipid-encapsulated SPIO nanoparticles exhibited no statistically significant differences in iron absorption at any tested concentration for a short exposure of 1 h or extended incubation of 24 h compared with the negative control (Fig. 2c). All lipid-coated SPIO nanoparticle groups exhibited an Fe concentration close to that of the negative control (0.002 ng). By contrast, the uncoated SPIO nanoparticles exhibited significantly greater uptake (two-tailed Student *t* test, *P* > 0.05) when the Fe concentration was higher than 0.05 mg/ml for 1-h incubation and 0.01 mg/ml for 24-h incubation (Fig. 2c). Therefore, the lipid-coated SPIO nanoparticles were used for antibody conjugation because of their superior nonfouling property.

To examine the biocompatibility of lipid-coated SPIO nanoparticles, the cell viability was evaluated using an MTT assay in U-87MG and U-251 GBM cells incubated with lipid-coated SPIO nanoparticles for 24 h at various concentrations. The results revealed that lipid-encapsulated SPIO nanoparticles are nontoxic to the tested GBM cells up to a concentration of 0.2 mg/ml (Fig. 2d).

### Synthesis of anti-EGFR antibody—functionalized targeted SPIO nanoparticles

**Fig. 3:**
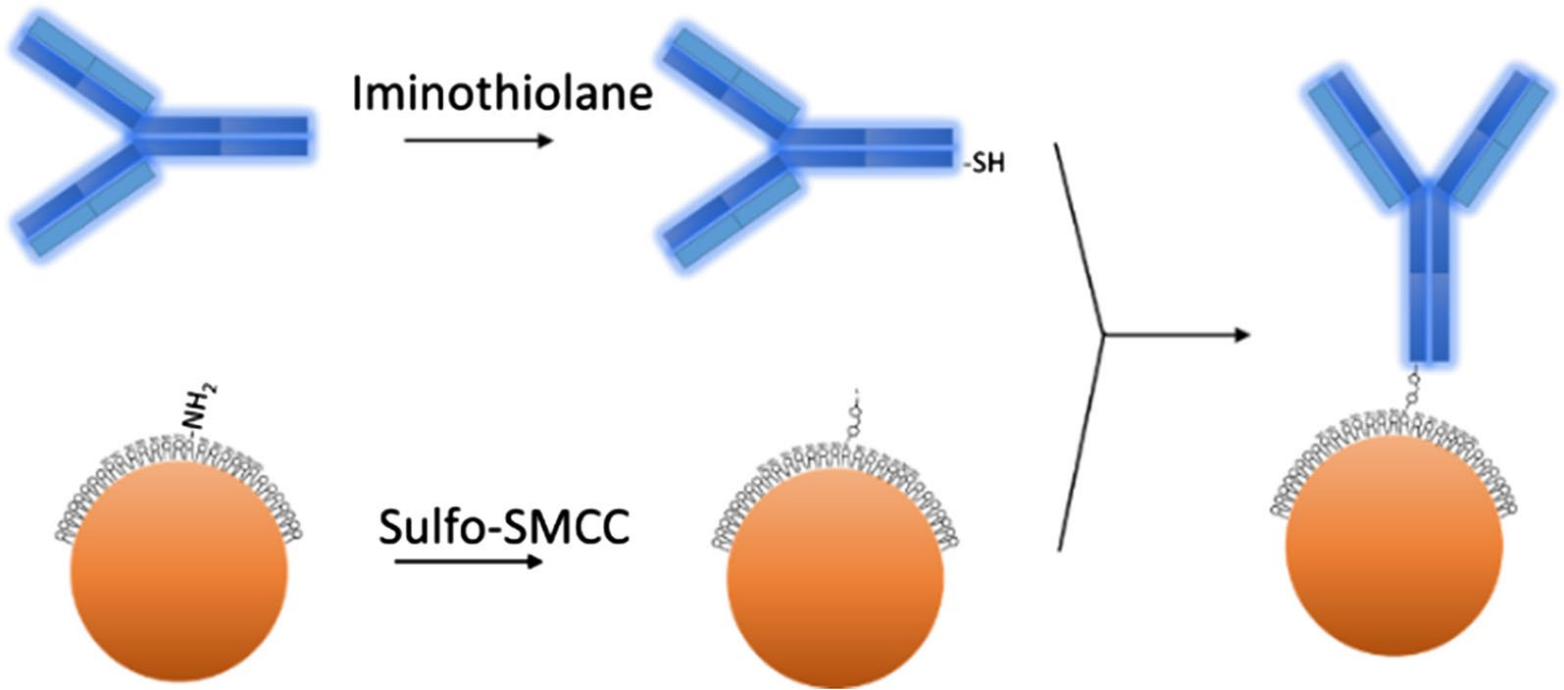
Schematic of anti-EGFR antibody cetuximab conjugated to lipid-encapsulated nonfouling SPIO nanoparticles. Maghemite nanoparticles were modified with maleimide through reaction with sulfo-SMCC; a thiolated antibody was prepared by treating cetuximab with iminothiolane. The maleimide-functionalized SPIO nanoparticles were mixed with the thiolated antibody solution to form antibody-conjugated SPIO nanoparticles. The lipid-coated surface of SPIO nanoparticles provides a nonfouling property

For targeting EGFR-expressing GBM cells, the anti-EGFR monoclonal antibody cetuximab was conjugated with the 10-nm lipid-encapsulated SPIO nanoparticle surface. A schematic of the conjugation progress is presented in Fig. 3. In brief, the amine-functionalized lipid-coated SPIO nanoparticles were reacted with sulfo-SMCC to obtain maleimide-functionalized SPIO nanoparticles. The anti-EGFR antibody cetuximab was treated with iminothiolane to form free thiol groups before spontaneous reaction with the maleimide-functionalized lipid-coated SPIO nanoparticles. This process produced EGFR-SPIO nanoparticles, as shown in Fig. 3. The coupling strategy enables direct robust covalent conjugation of the antibody to SPIO nanoparticles, which provides greater stability than that of the noncovalent immobilization approaches in biological conditions [35].

### Characterization of EGFR-SPIO nanoparticles

**Fig. 4:**
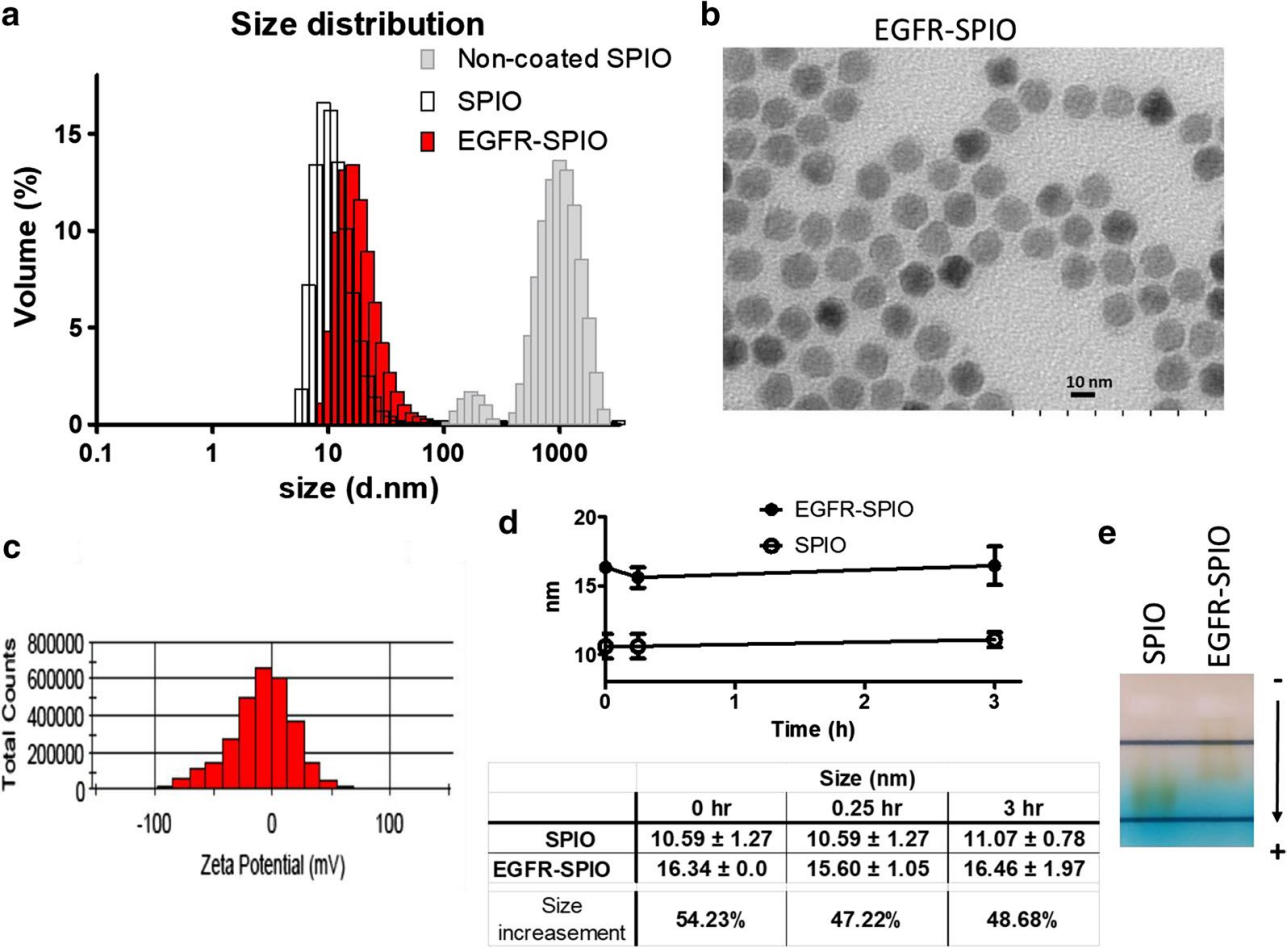
Characterization of physical properties of EGFR-SPIO nanoparticles and validation of antibody-SPIO conjugation. **a** Size distribution of EGFR-SPIO, unconjugated lipid SPIO, and non-coated SPIO nanoparticles were determined through DLS measurement. The size of EGFR-SPIO peaked at 16.34 ± 0.0 nm on volume distribution graph and that of unconjugated lipid SPIO nanoparticles peaked at 10.59 ± 1.27 nm. The non-coated SPIO aggregated and measurement peaked above 1000 nm. **b** TEM image of EGFR-SPIO nanoparticles. Scale bar, 10 nm. **c** Zeta potential statistics graph of EGFR-SPIO nanoparticles. The zeta potential of EGFR-SPIO nanoparticles peaked at − 9.24 ± 0.43 mV. **d** The stability of EGFR-SPIO conjugates was evaluated by measuring the variation of size through DLS. The indicated nanoparticles diluted in PBS (1:12) were incubated for 0, 0.25, and 3 h at room temperature before subjecting to DLS size measurement. The peak of volume distribution from each time point was measured and averaged, and the % of volume incensement after the cetuximab conjugation on lipid SPIO was calculated. The results indicate that EGFR-SPIO nanoparticles were stable over the observation period at room temperature. **e** Band shift assay of EGFR-SPIO conjugates. Unconjugated lipid SPIO and EGFR-SPIO nanoparticles were subjected to electrophoresis on 1% agarose gel in TAE buffer. Arrowhead, migration direction; −, cathode; +, anode

The synthesized EGFR-SPIO nanoparticles were characterized using DLS, zeta potential, and TEM image assays to confirm the conjugation and stability. The DLS measurement indicated that the size of EGFR-SPIO nanoparticles was 16.34 ± 0.0 nm, a 54.30% increase compared with the size of 10.59 ± 1.27 nm for unconjugated lipid SPIO nanoparticles (Fig. 4a). The increased size suggests that the conjugation of the antibody cetuximab to SPIO was successful. The DLS measurement of non-coated SPIOs peaked above 1000 nm, suggests these nanoparticles may aggregate. To confirm the size of nanoparticles, the diameter of nanoparticles was measured by TEM (Additional file 1: Figure S2); the diameter of lipid-coated SPIOs measured 12.31 ± 0.66 nm, and EGFR-SPIOs measured 13.07 ± 0.71 nm. The non-coated SPIOs synthesized by coprecipitation method was un-homogenous and aggregated on the TEM images, and the diameter of it measured 7.25 ± 2.43 nm. The TEM images of conjugated EGFR-SPIO nanoparticles are presented in Fig. 4b. The zeta potential of EGFR-SPIO nanoparticles peaked at − 9.24 ± 0.43 mV (Fig. 4c), whereas the zeta potential of lipid-coated SPIO nanoparticles peaked at − 5.42 ± 0.9 mV. The decrease in the zeta potential indicates that the surface of SPIO nanoparticles had been modified by the antibody conjugation procedures. Moreover, to confirm the conjugation between SPIO nanoparticles and the antibody, purified SPIO nanoparticles were subjected to a dot blot assay to verify the presence of the antibody on them. As shown in Additional file 1: Figure S1, EGFR-SPIO nanoparticles exhibited robust ECL signals in the dot blot assay used for detecting human antibodies. No signal was observed in the bare lipid SPIO groups.

Moreover, the stability of EGFR-antibody conjugated SPIO nanoparticles was evaluated (Fig. 4d). Size variations of EGFR-SPIO and lipid SPIO nanoparticles were observed at 0, 0.25, and 3 h in PBS at pH 7.4. The size of EGFR-SPIO nanoparticles was 10.59 ± 1.27, 10.59 ± 1.27, and 11.07 ± 0.78 nm at 0, 0.25, and 3 h, respectively, and the size of lipid SPIO nanoparticles was 16.34 ± 0.0, 15.60 ± 1.05, and 16.46 ± 1.97 nm at 0, 0.25, and 3 h, respectively (Fig. 4d). No statistically significant differences were observed during the observation period. Notably, the size remained stable over the observation period in both the EGFR-SPIO and bare lipid SPIO nanoparticle groups, suggesting that no antibody dissociation was detected over the observation period (Fig. 4d). The reprehensive DLS stability graphs of nanoparticles at physiological conditions include PBS, cell culture medium with 10% fetal bovine serum (FBS) or in pure FBS are shown in Additional file 1: Figure S3A. The DLS of lipid-SPIOs or EGFR-SPIOs maintain stable in the 3-h observation period. In contrast, the non-coated SPIOs aggregated when presented in the PBS (Additional file 1: Figure S3A, B), while the lipid-coated SPIOs or EGFR-SPIOs maintain dispersion in the same condition. In addition, to further confirm antibody–SPIO nanoparticle conjugation, the specimens were subjected to a gel electrophoresis assay. As shown in Fig. 4e, the migration rate of EGFR-SPIO nanoparticles was significantly lower than that of lipid SPIO nanoparticles, indicating that the net-charge-to-volume ratio was altered after the antibody-SPIO nanoparticle conjugation procedures.

### Evaluation of the targeting specificity of EGFR-SPIO nanoparticles to EGFR-expressing GBM cells

**Fig. 5:**
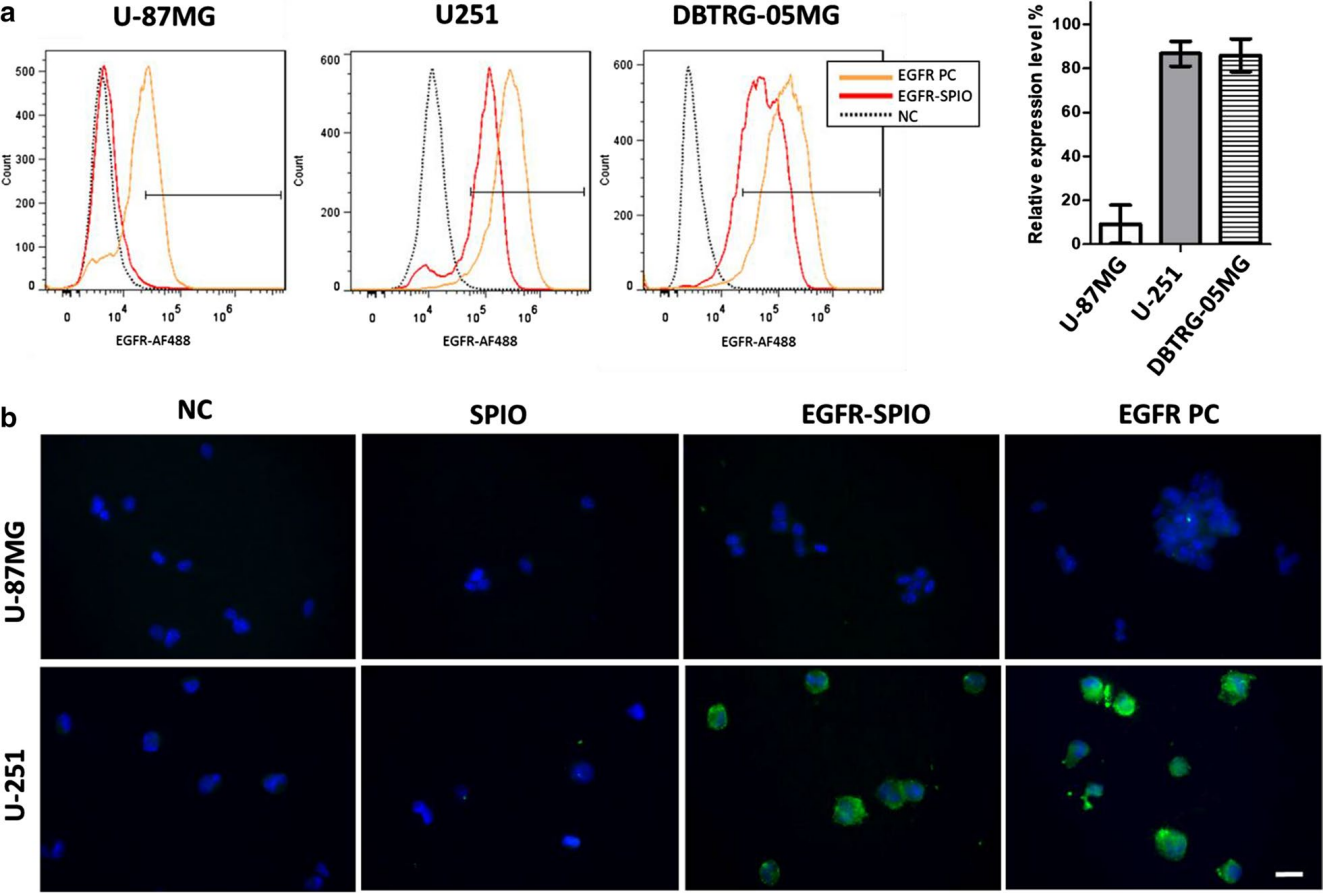
Evaluation of EGFR-SPIO nanoparticle targeting efficiency in GBM cell lines. **a** The targeting efficiency of EGFR-SPIO nanoparticles was quantified using flow cytometry. Resuspended U-87MG, U-251, and DBTRG-05MG GBM cells were subjected to flow cytometry analysis with EGFR-SPIO nanoparticles or the anti-EGFR antibody and goat anti-human AF-488 antibody. **b** Evaluate targeting specificity of EGFR-SPIO nanoparticles by IF staining. U-87MG and U-251 cells were fixed and incubated with empty media (negative control, NC), lipid SPIO nanoparticles, EGFR-SPIO nanoparticles, or the cetuximab for positive control (EGFR PC). After PBS wash, the specimens were incubated with goat anti-human AF-488 antibody to detect the presence of cetuximab. Green, cetuximab; blue, DAPI. Scale bar, 20 µm

To evaluate the EGFR targeting specificity of the EGFR antibody after conjugation with SPIO nanoparticles, cell affinity experiments were conducted using the GBM cell lines U-87MG (EGFR^low^), U-251 (EGFR^high^), and DBTRG (EGFR^high^). The flow cytometry analysis results (Fig. 5a) revealed that compared with the EGFR antibody control, the synthesized EGFR-SPIO nanoparticles selectively bound to the U-251 and DBTRG-05MG; 86.67 and 85.95% of the cells were detected as EGFR positive by using EGFR-SPIO nanoparticles. By contrast, EGFR-SPIO nanoparticles had a negligible affinity toward U-87MG, which has a lower EGFR expression level. As few as 9.19% of U-87MG cells were labeled by EGFR-SPIO nanoparticles.

Furthermore, the targeting specificity of EGFR-SPIO nanoparticles was evaluated using an IF labeling technique. The IF results revealed markedly strong green fluorescence signals specifically on the EGFR^high^ expresser U-251 (Fig. 5b), indicating the affinity and binding magnitude of the EGFR-SPIO nanoparticles to EGFR-expressing cells. By contrast, no green fluorescence signal was observed in the EGFR^low^ expresser U-87MG, indicating that nonspecific interaction was not detected in the EGFR-SPIO nanoparticles. No signal was detected in the lipid SPIO nanoparticle controls. The flow cytometry and IF results suggested that cetuximab retained its affinity specifically toward the EGFR after being conjugated to SPIO nanoparticles.

### Validation of the presence of targeted SPIO nanoparticles on EGFR-expressing GBM cells through Prussian blue staining and TEM

**Fig. 6:**
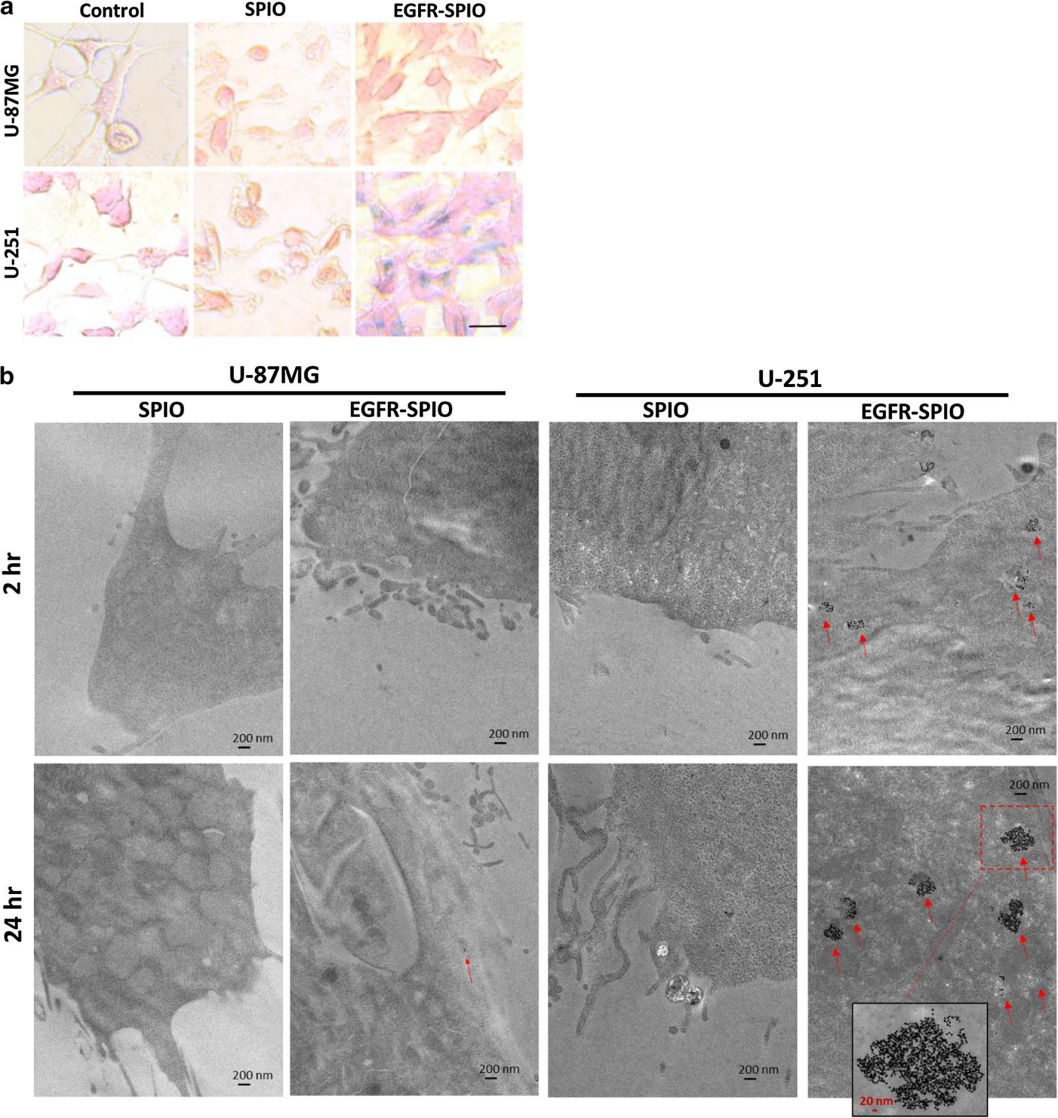
Evaluation of the presence of SPIO nanoparticles in GBM cell lines. **a** Prussian blue staining for SPIO nanoparticles in GBM cell lines. U-87MG and U-251 cells were incubated with control media, media containing unconjugated lipid SPIO nanoparticles or EGFR-SPIO nanoparticles (100 ng/ml) for 24 h before Prussian blue and nuclear fast red staining. Scale bar, 20 µm. **b** Lipid SPIO or EGFR-SPIO nanoparticle sorption was detected in U-87MG or U-251 MG cells through TEM. The indicated GBM cells were exposed to EGFR-SPIO or unconjugated lipid SPIO nanoparticle at 0.1 mg/ml for 2 or 24 h before subjecting them to TEM. 20,000× magnification; scale bar, 200 nm; arrowhead, SPIO cluster; red dashed-line box, area with 50,000× magnification; scale bar, 20 nm

To confirm the presence of targeted SPIO nanoparticles on EGFR^high^ GBM cells, Prussian blue staining was performed to detect the presence of iron oxide on U-87MG and U-251 GBM cells incubated with EGFR-SPIO or lipid SPIO nanoparticles for 24 h. Consistent with the IF staining results, Prussian blue staining detected blue signals only in the EGFR^high^ expresser U-251 incubated with EGFR-SPIO nanoparticles, which is direct evidence of the presence of iron oxide nanoparticles in these cells (Fig. 6a). By contrast, in the EGFR^low^ U-87MG cells, no Prussian blue signal was detected when the cells were incubated with EGFR-SPIO nanoparticles. No iron uptake was detected in U-87MG or U-251 cells incubated with SPIO nanoparticle controls in the same condition.

Furthermore, to validate the cellular localization of EGFR-SPIO nanoparticles and confirm the uptake of targeted nanoparticles after treatment, the presence of SPIO nanoparticles on U-87MG or U-251 cells treated for 2 or 24 h was detected using TEM. In the TEM images of SPIO or EGFR-SPIO nanoparticle-treated EGFR^low^ U-87MG cells, only sparse nanoparticles were detected in the cytoplasmic region after either 2- or 24-h treatment (Fig. 6b). By contrast, electron-dense SPIO nanoparticles were detected on the cell surface and in the endosome of EGFR^high^ U-251 cells treated with EGFR-SPIO nanoparticles for 2 h. Moreover, the SPIO concentration in the endosome increased only in EGFR-SPIO nanoparticle-treated U-251 cells. No SPIO uptake into the endosome was detected in U-251 cells treated with bare lipid SPIO nanoparticles for 2 or 24 h. In each SPIO-containing endosome, more than 40 particles were detected in the condensed region of TEM sections at a magnification of 20,000× (Fig. 6b).

### MR T_2_ relaxation time and fitting curve of EGFR-SPIO nanoparticle phantoms

**Fig. 7:**
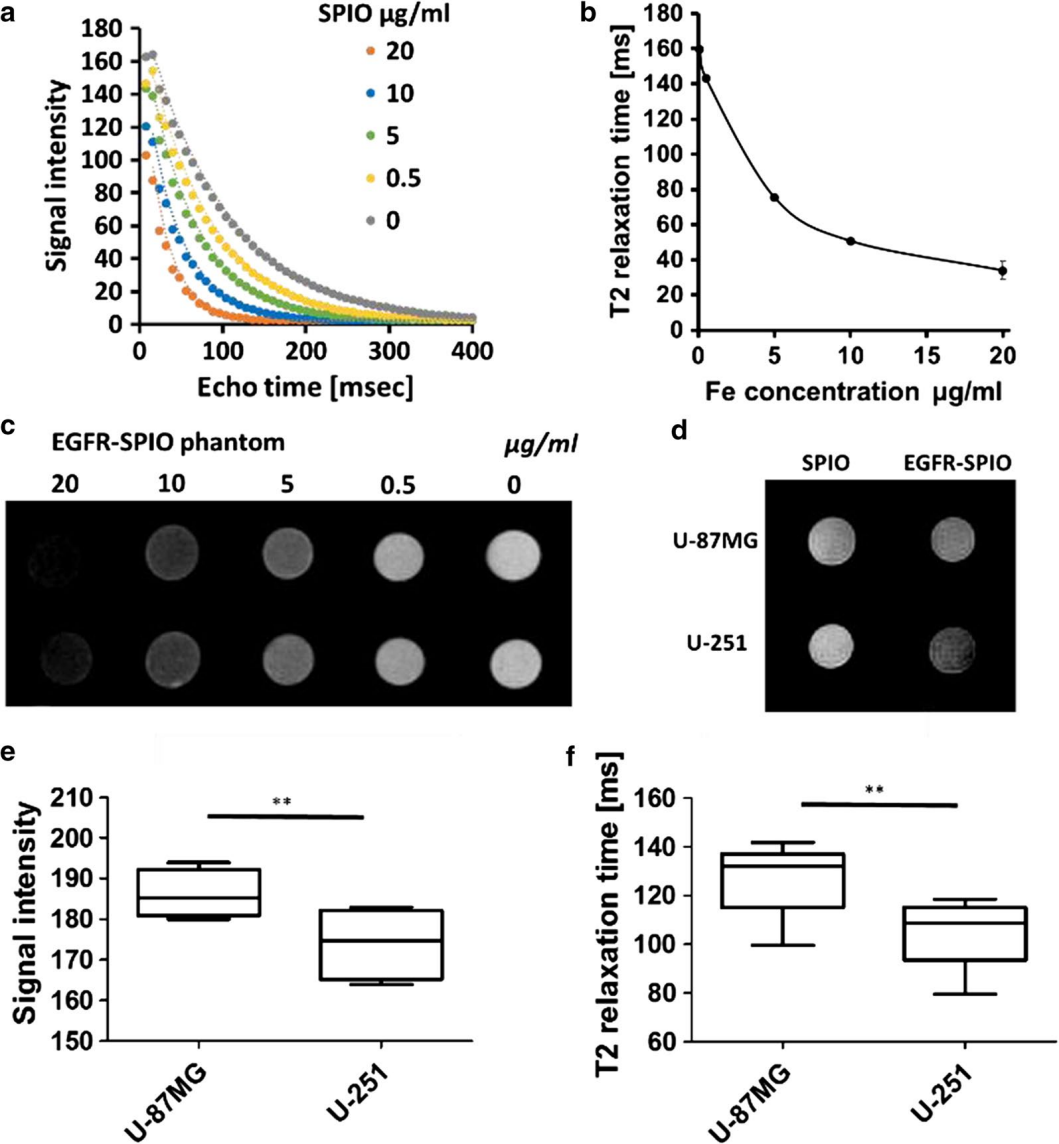
Detection of EGFR-SPIO nanoparticle binding in U-87MG and U-251 cells in vitro. **a** Echo time curve fitting of EGFR-SPIO nanoparticle phantoms in various known concentrations was measured using 7T MRI. **b** T_2_ relaxation time of EGFR-SPIO nanoparticles in known Fe concentrations (*n* = 3 for each concentration). **c** Corresponding signal intensity images of EGFR-SPIO standards measured using T_2_-weighted MR. Darkest to brightest, 20, 10, 5, 0.5, and 0 μg/ml. **d** T_2_-weighted in vitro images of EGFR-SPIO nanoparticle-treated U-87MG and U-251 cells. Cells were incubated with EGFR-SPIO nanoparticles at 0.1 mg/ml at 37 °C for 2 h. A reduction in T_2_ signal intensity was observed in EGFR^high^ U-251 cells compared with EGFR^low^ U-87MG cells. **e** Signal intensity of EGFR-SPIO nanoparticle-treated U-87MG and U-251 cells in box plot. **f** T_2_ relaxation time of EGFR-SPIO nanoparticle-treated U-87MG and U-251 cells in box plot

To evaluate the feasibility of using lipid-encapsulated EGFR-SPIO nanoparticles for EGFR-positive cell detection through MRI, EGFR-SPIO nanoparticle phantom scans were performed to investigate the MRI signal intensity and response to T_2_ relaxation time at various concentrations. The signal intensity and T_2_ relaxation time of EGFR-SPIO nanoparticles suspended in agarose gel with known Fe concentrations of 20, 10, 5, 0.5, and 0 μg/ml were measured using 7T MRI. The T_2_ fitting curve of EGFR-SPIO nanoparticles exhibited a correlation between the signal intensity and nanoparticle concentration at TE values ranging from 8 to 100 ms (Fig. 7a). Moreover, the EGFR-SPIO nanoparticles showed a decrease in MR T_2_ relaxation time with an increase in Fe concentration (Fig. 7b). The T_2_ relaxation time of EGFR-SPIO nanoparticles was 159.61 ms at 0.1 μg/ml and decreased to 33.84 ms when the Fe concentration was increased to 20 μg/ml. The corresponding T_2_-weighted MR signal intensity images of EGFR-SPIO phantoms were shown on Fig. 7c. The strong correlations of the EGFR-SPIO nanoparticle concentration with the MRI T_2_ relaxation time and signal intensity suggest that the concentration of the synthesized EGFR-SPIO nanoparticles can be distinguished through MRI.

### Identification of EGFR-positive GBM cells through in vitro MRI

To evaluate whether lipid-encapsulated EGFR-SPIO nanoparticles could be used for EGFR-positive cell detection, EGFR^high^ U-251 or EGFR^low^ U-87MG cells were incubated with EGFR-SPIO or SPIO nanoparticles before subjecting them to molecular MR image detection. The MRI signal intensity decreased in EGFR^high^ U-251 cells incubated in media containing EGFR-SPIO nanoparticles compared with the intensity in the SPIO control group (Fig. 7d). Furthermore, both the signal intensity and T_2_ relaxation time could be used for distinguishing EGFR^high^ U-251 from EGFR^low^ U-87MG GBM cells. The EGFR-SPIO nanoparticle-treated U-251 cells exhibited a significant decrease in MRI signal intensity compared with the U-87MG cells in the same condition (Fig. 7e). Moreover, the EGFR-SPIO nanoparticle-treated U-251 cells exhibited a prominent decrease in T_2_ relaxation time compared with the U-87MG cells, which suggests that the EGFR-SPIO nanoparticles selectively targeted EGFR^high^ U-251 cells (Fig. 7f). From the T_2_ relaxation time results, the iron concentrations were estimated as 1.55 ± 0.96 and 3. 02 ± 0.89 μg/ml in EGFR-SPIO nanoparticle-treated U-87MG cells and U-251 cells, respectively. These results indicate that the synthesized EGFR-SPIO nanoparticles can be used for identifying EGFR-positive GBM cells in vitro.

## Discussion

Targeted MRI nanoparticles have demonstrated high potential for noninvasive detection of disease status; however, the use of such particles in cancer subtyping or tumor marker detection is still under investigation. Moreover, the application of targeted nanoparticles is still hindered by the nonspecific uptake of nanoparticles by macrophages, Kupffer cells, or the kidney. Various approaches have been adopted to resolve this issue, including conventional dextran or polyethylene glycol surface modifications of SPIO nanoparticles. Recently, coating an artificial surface with a lipid to mimic the cell surface membrane has been explored, and such coatings have a promising antifouling property in biological conditions [29]. However, the current SPIO nanoparticle fabrication strategy did not fully explore the potential use of the lipid-coating surface to reduce non-specific binding validated in other systems.

In this study, we demonstrated a method of fabricating lipid-encapsulated SPIO nanoparticles with the anti-EGFR antibody cetuximab for in vitro MRI analysis. Such nanoparticles can specifically target EGFR^high^ U-251 GBM cells and generate signals distinguishable from those of EGFR^low^ U-87MG cells in MRI analysis in vitro. In addition, the lipid coating on the SPIO nanoparticles exhibits a potent nonfouling property that efficiently suppresses the nonspecific sorption of SPIO nanoparticles by EGFR^low^ GBM cells. This could reduce system noise in MRI, thus preventing false-positive signals. Our results reveal that lipid-encapsulated SPIO nanoparticles suppress nonspecific sorption in GBM cells, which is in agreement with previous observations regarding such modified materials [29, 30].

Conjugating cetuximab to SPIO nanoparticles enables specific targeting toward the EGFR (Figs. 5, 6, 7), and the antibody-SPIO nanoparticle conjugation process in this study did not alter the antibody’s affinity, as confirmed using flow cytometry analysis, IF staining, Prussian blue staining, and TEM. Furthermore, the synthesized EGFR-SPIO nanoparticles were successfully applied for in vitro MRI analysis. According to the T_2_ relaxation time and mean intensity of the MR images, EGFR^high^ U-251 cells were distinguished from EGFR^low^ U-87MG cells in vitro, which suggests the potential use of this system in noninvasive EGFR status detection for brain tumors. According to our review of relevant literature, this study is the first to describe the conjugation of cetuximab to lipid-coated SPIO nanoparticles.

Although the monoclonal antibody-guided targeted SPIO nanoparticles have great potential for application in noninvasive EGFR detection by using molecular MRI, a few obstacles must be addressed before performing a thorough in vivo analysis. The blood–brain barrier (BBB) formed by the microvascular endothelial cells of the brain represents a great challenge for nanoparticles that target the central nervous system (CNS). The BBB is known to impede macromolecules in the circulation from entering the CNS. However, few mechanisms through which macromolecules can cross the BBB have been explored. First, the kinetics of BBB transport is affected by the size of SPIO nanoparticles. As demonstrated in Sonavane’s study, nanoparticles smaller than 50 nm can passively permeate the BBB and accumulate in the brain [36]. In addition, Shilo reported that 20 nm is the optimal size for maximizing the free surface area of nanoparticles that enter the brain cells [37]. In this study, the core size of the SPIO nanoparticles was 10 nm, and the average size of the EGFR-SPIO nanoparticles was 16.34 nm, which is within the range of high BBB permeability. Second, surface chemistry can also influence the BBB permeability. Lipid-soluble nanoparticles have an increased membrane transport ratio, which facilitates BBB crossing [38]. In addition, Gabriel demonstrated that solid lipid nanoparticles loaded with iron oxide can cross the BBB and accumulate in the brain with long-lasting kinetics [39]. Fenart et al. demonstrated that BBB crossing by lipid-coated ionically charged nanoparticles was three- or fourfold higher compared with that by uncoated particles [40]. Moreover, their study showed that lipid-coated nanoparticles did not impair BBB integrity, and BBB crossing was achieved through transcytosis without any degradation [40]. These studies indicate that the lipid-encapsulated SPIO nanoparticles in the present study may be a promising vehicle for molecular imaging studies that target molecules behind the BBB. In this study, SPIO nanoparticles were encapsulated in phospholipid derivatives for providing a functionalized surface for conjugation and potentially promoting BBB permeability.

With the advances in targeted therapies, a great challenge for cancer treatment is identifying signatures of the tumor markers for each individual. Conventionally, tumor markers such as the EGFR expression level and copy number change are identified through IHC staining, PCR, or FISH analysis with specimens obtained from surgical resection or biopsy. Tumor markers for cancer subtyping are applicable for treatment plan development, disease status monitoring, and disease prognosis. Although the demand for accurate molecular diagnosis is increasing, a systematic, noninvasive strategy for evaluating tumor markers in highly heterogeneous tumor environments is still lacking. The T_2_ relaxation time of MR images obtained using the EGFR-SPIO nanoparticles constructed in this study successfully distinguished EGFR^high^ from EGFR^low^ GBM cells, suggesting the potential use of this targeted lipid-encapsulated SPIO nanoparticle system for in vivo detection in the future. This is a promising approach for identifying tumor markers in brain cancer, particularly when conventional biopsy methods may not be desirable.

## Conclusion

In this study, a rapid and noninvasive method of molecular MRI was established to identify EGFR-positive GBM by using novel EGFR-SPIO nanoparticles with a lipid coating. The results indicate that T_2_ MR images from EGFR-SPIO nanoparticles can be used for identifying EGFR-positive GBM in vitro. Efforts to determine the in vivo detection efficiency of this approach are under way.

## Additional file

**Additional file 1. Figure S1:** Validation of anti-EGFR-antibody conjugation to SPIO nanoparticles using dot-blot assay. **Figure S2**: Measure the diameter of nanoparticles by TEM images. **Figure S3**: Characterization of iron oxide nanoparticles in physiological conditions.

## Abbreviations

EGFR: epidermal growth factor receptor
FBS: fetal bovine serum
GBM: glioblastoma multiforme
IF: immunofluorescence
SPIO: superparamagnetic iron oxide
MRI: magnetic resonance imaging
MR: magnetic resonance
MEM: minimum essential media
TEM: transmission electron microscopy
T_2_: transverse relaxation time
TE: echo time
TR: repetition time
