# Supplementary material for: Identification of epidermal growth factor receptor-positive glioblastoma using lipid-encapsulated targeted superparamagnetic iron oxide nanoparticles in vitro
Source: J Nanobiotechnology. 2017 Nov 22;15:86. doi: 10.1186/s12951-017-0313-2 (PMC5700523; doi:10.1186/s12951-017-0313-2)
Supplement: Supplementary file 1 — Additional file 1. Figure S1: Validation of anti-EGFR-antibody conjugation to SPIO nanoparticles using dot-blot assay. Figure S2: Measure the diameter of nanoparticles by TEM images. Figure S3: Characterization of iron oxide nanoparticles in physiological conditions. [file 12951_2017_313_MOESM1_ESM.docx]

## Additional file 1


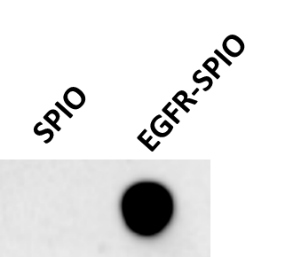


**Figure S1.** **Validation of anti-EGFR-antibody conjugation to SPIO nanoparticles using dot-blot assay.**

In order to evaluate the presence of conjugated anti-EGFR-antibody on SPIO nanoparticles, EGFR-SPIO or bare lipid-SPIO nanoparticles were subjected to dot-blot analysis. Two μl of the indicated nanoparticles were probed on a PVDF membrane and incubated with the goat anti-human HRP secondary antibody. After that, the presence of the humanized anti-EGFR-antibody was visulaized by a chemiluminescence assay.

**
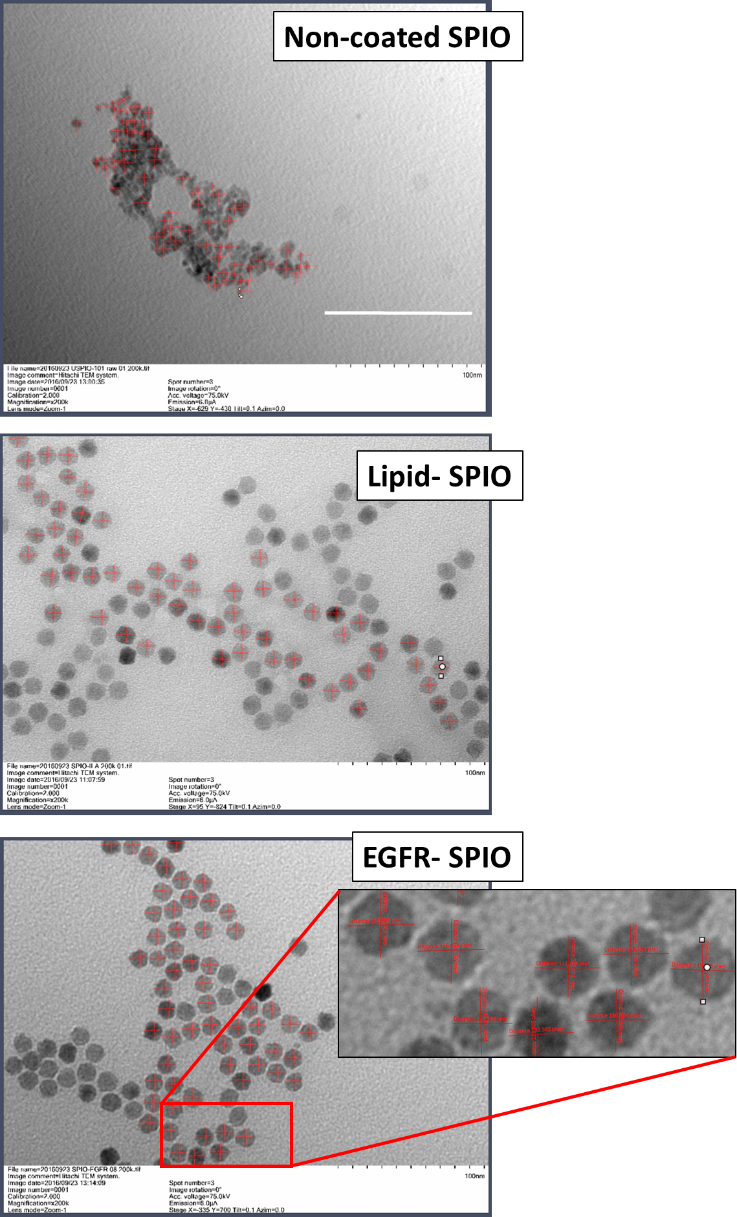
**

**Figure S2. Measure the diameter of nanoparticles by TEM images.**

The diameter of bare SPIOs and EGFR-SPIOs were measured from 200K enlarged TEM images. The scale of the image was first calibrated against the scale bar from the TEM images in the Zen image analysis software before diameter measurement. After calibrated, the diameter from both the horizontal and vertical axis of each nanoparticle was measured across the TEM image. Over 100 measurements were performed on >50 nanoparticles for each type of nanoparticle. The size of non-coated SPIOs, lipid-SPIOs, and EGFR-SPIOs are measured 7.25 ±2.43 nm, 12.31 ±0.66 nm, and 13.07 ±0.71 nm respectively.

A
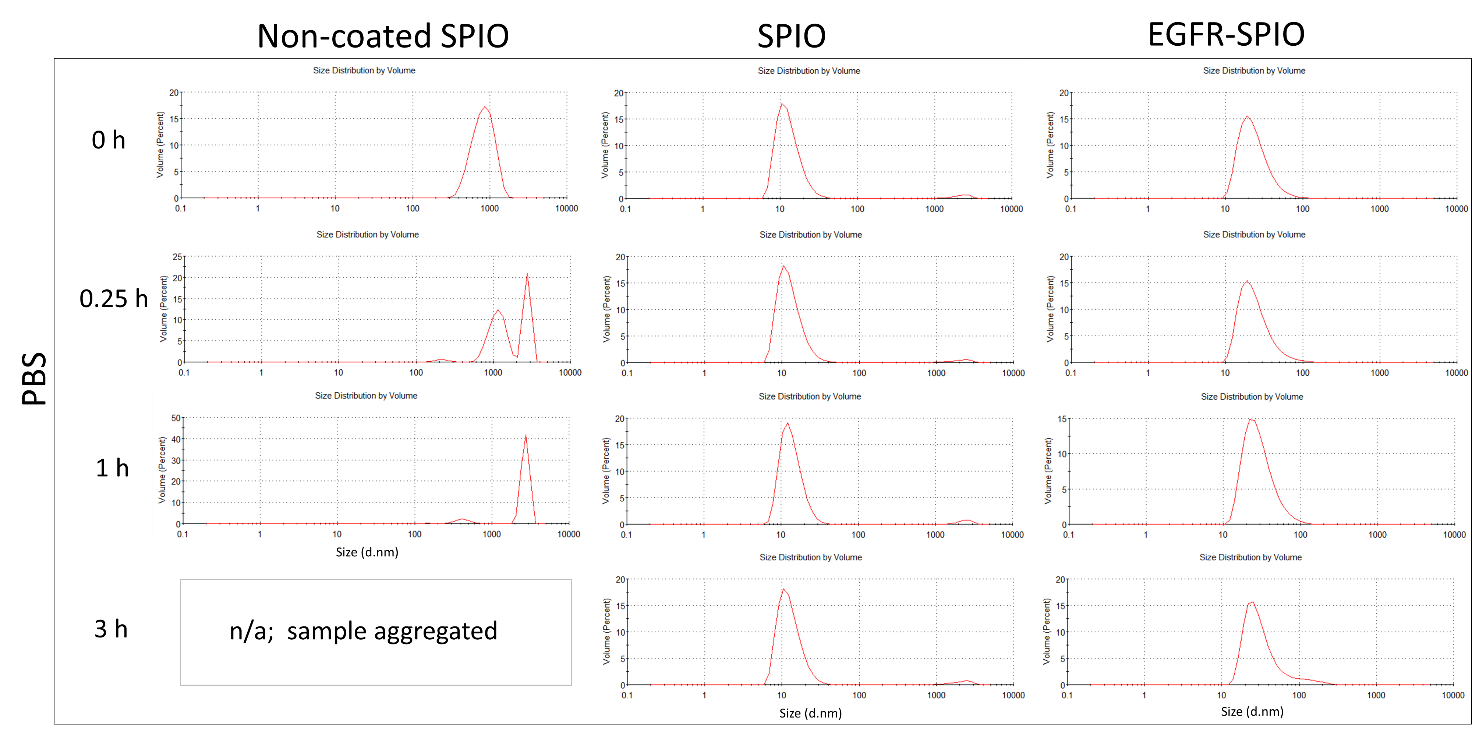

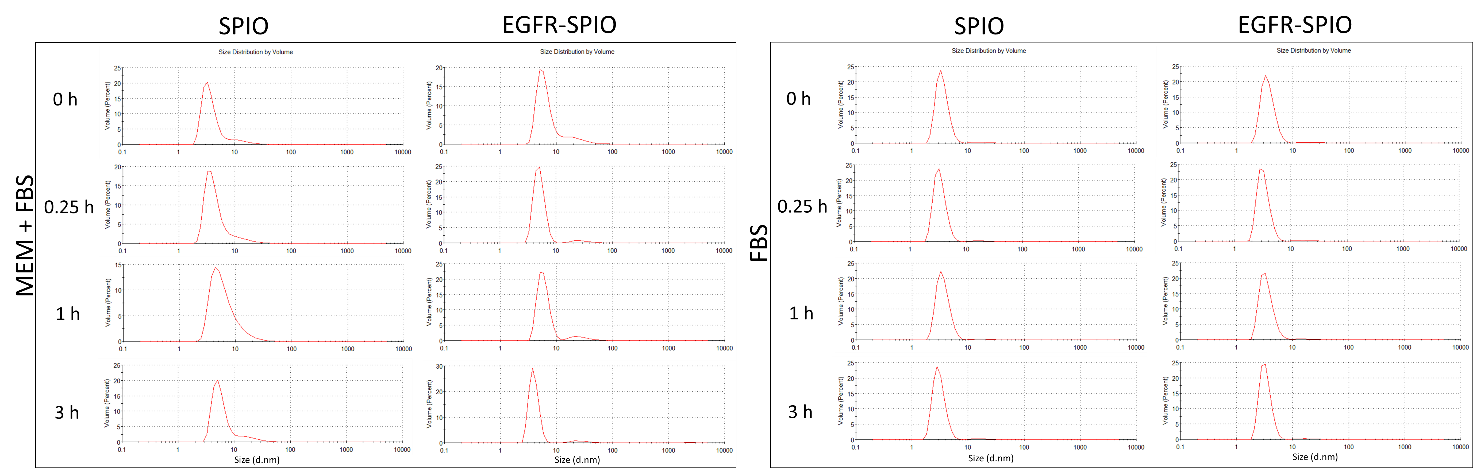


B


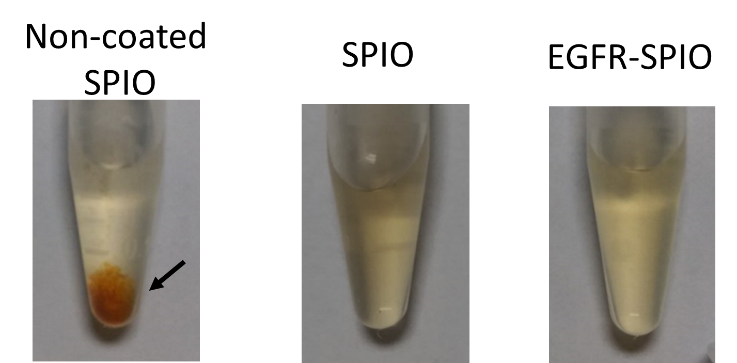


**Figure S3. Characterization of iron oxide nanoparticles in physiological conditions.**

A) Evaluate nanoparticles stability of non-coated SPIO, lipid-coated SPIO or EGFR-SPIO in physiological conditions by DLS measurement. The indicated nanoparticles were incubated with PBS, minimal essential medium (MEM) + 10% FBS, or FBS for 0, 0.25, 1 and 3 h at room temperature. The DLS volume distribution results suggest the synthesized EGFR-SPIO nanoparticles were stable under tested physiological conditions during the observation period. The non-coated SPIO- PBS 3 h group is not available due to sample aggregation. n/a, not available.

B) Images of iron oxide nanoparticles when presented in PBS for 3 h. The non-coated SPIOs aggregated after mixed with PBS and precipitated (arrowhead), while lipid- coated SPIOs or EGFR-SPIOs maintain dispersion in PBS throughout the test period.
